# Effectiveness of neutralizing antibody cocktail in hemodialysis patients: a case series of 20 patients treated with or without REGN-COV2

**DOI:** 10.1007/s10157-021-02151-3

**Published:** 2022-02-18

**Authors:** Shigehisa Arikawa, Kazuhito Fukuoka, Keitaro Nakamoto, Rie Kunitomo, Yuki Matsuno, Teppei Shimazaki, Takeshi Saraya, Takahisa Kawakami, Mitsumasa Kishimoto, Yoshinori Komagata, Daisuke Kurai, Haruyuki Ishi, Shinya Kaname

**Affiliations:** 1 Department of Nephrology and Rheumatology, Kyorin University School of Medicine, 6-20-2 Shinkawa, Mitaka-shi, PO 181-8611, Tokyo, Japan; 2 Department of Respiratory Disease, Kyorin University School of Medicine, 6-20-2 Shinkawa, Mitaka-shi, Tokyo, Japan; 3 Department of General Medicine, Kyorin University School of Medicine, 6-20-2 Shinkawa, Mitaka-shi, Tokyo, Japan

**Keywords:** COVID-19, REG-COV2 (a neutralizing antibody cocktail), Hemodialysis, Pneumonia, End-stage kidney disease

## Abstract

The number of patients with SARS-CoV-2 infection continues to increase, and it has become a global pandemic. Although there is an urgent need to establish an effective treatment, the medication available for dialysis patients has been limited. An antibody cocktail containing two SARS-CoV-2-neutrarizing antibodies, REGN-COV2 has been granted special approval for COVID-19 in Japan, since July 2021, and this intravenous preparation can be used for dialysis patients. At our hospital, we had 22 hemodialysis patients with COVID-19, and five of them were treated with REGN-COV2. On admission, four of the five patients had moderate disease (pneumonia but O_2_ inhalation) and one patient had mild disease (not having pneumonia). The mean duration of hospitalization treated with REGN-COV2 was 10.2 ± 2.86 days (mean ± SD), which was less than half, compared to patients untreated of similar severity on admission (22.12 ± 15.5). The time to fever resolution was average 7 days, and no cases progressed to severe illness or death. Among these patients, no obvious adverse reactions were shown. Although more studies with a larger number of patients could be needed for a rigorous evaluation of the effect, our result suggests that REGN-COV2 may be safe and having the possibilities in preventing severe disease in hemodialysis patients. Given the difficulty in securing inpatient beds tend to be in short supply, the strategy combined with neutralizing antibody could be beneficial for end-stage kidney disease (ESKD) patients with hemodialysis who are at high risk of severe disease.

## Background

SARS-CoV-2, an emerging infectious pathogen, which presents with a severe acute respiratory syndrome, was first identified in December 2019, and has become a worldwide pandemic [1]. Currently, remdesivir, dexamethasone, tocilizumab, and baricitinib, have been approved for the treatment of SARS-CoV-2 infection, COVID-19 [2–5]. However, the usage of some of these medications is limited in patients with end-stage kidney disease (ESKD) on hemodialysis. In particular, remdesivir is used for mild to moderate coronavirus infections, but it is not available for use in patients with renal failure, which means that these patients are at high risk of severe disease but cannot receive therapeutic medication until they need oxygen. Furthermore, the number of hemodialysis patients is much smaller than that of the general population, which makes it difficult to clarify the effective strategy of COVID-19 treatment. Recent day, an antibody cocktail containing two SARS-CoV-2-neutrarizing antibodies (casirivimab/imedevimab) (REGN-COV2) was approved and was granted special approval in Japan. This medication is indicated for mild to moderate cases that do not require oxygen inhalation and used to prevent the disease from progressing. It is eligible to administer in ESKD patients with hemodialysis, therefore it is expected to become a key drug for preventing COVID-19 deteriorate to severe disease in these patients. We treated hemodialysis patients with REGN-COV2 in our hospital. Here, we would report its treatment results as a case series and compare with the patients with the same severity.

## Subjects and methods

### Study design and subjects

This is a case–control study to evaluate the efficacy of REGN-COV2, and retrospectively analyzed the clinical course and laboratory data of dialysis patients with COVID-19. The subjects were adult patients on maintenance hemodialysis admitted due to COVID-19 to Kyorin University hospital from December 2019 to September 2021. The comparison of physical finding, laboratory data and clinical course between patients who were treated with REGN-COV2 and those who were not, among the patients who were in the same severity stage. The indications for REG-COV2 treatment in Japan are patients with disease severity up to moderate I within 7 days of onset (patients who do not require oxygen administration with or without pneumonia at the time of hospitalization) and patients at risk of severe COVID-19 (elderly, obesity, obstructive pulmonary disease, cancer patients, chronic kidney disease, hyperlipidemia, smoking, pregnancy, and patients on immunosuppressive drugs due to transplantation). Therefore, all patients on dialysis are risk holders (CKD G5d) and met the induction criteria for REGN-COV2. Patients who already had severity II or higher (patients requiring oxygen administration) on admission were excluded from the analysis.

### REGN-COV2 administration procedure

The administration was performed in accordance with the instruction provided by the pharmaceutical company. Briefly, immediately before administration, 600 mg each of *casirivimab* and *imdevimab* were withdrawn from the vials, added to 100 ml of saline, and infused intravenously over 60 min. Although it was administered intravenously to dialysis patients, the dose and timing of administration was the same as for non-dialysis patients.

### Statistical analysis

All data were analyzed with SPSS^®^ 24.0 (IBM, Inc., Chicago, IL, USA). Data have been presented as the mean ± standard deviation. The *t test* was used for comparisons between two group. *P* values less than 0.05 were regarded as statistically significant.

## Results

### Case presentation

Case 1 is a 55-year-old man, who visited our hospital due to fever and dyspnea. He had a fever of 37.5 °C and did not improve. In the day four from the onset of initial symptom, he was admitted to our hospital because of COVID-19 diagnosed with the positive in PCR test of SARS-CoV-2.0. He had been on hemodialysis for almost nine years due to end-stage kidney disease (ESKD) caused by diabetic nephropathy (DMN). He had a history of duodenal ulcer and vitreous hemorrhage in the left eye and had hypertension. He had a smoking history and has not been vaccinated yet. On admission, his blood pressure was 184/112 mmHg, heart rate 62 bpm, respiratory rate was normal and showed no abnormality in oxygen saturation (SpO_2_ 98%). Although there were no significant abnormalities except for high fever (39.2 °C) on the physical examinations, Chest CT scan showed the patchy ground glass opacities (GGO) bilaterally and a consolidation with air bronchogram were found in his left lower lung (Fig. 2a). Considering the possibility of bacterial coinfection, 1.0 g per day of ceftriaxone sodium was begun with REGN-COV2 on the second day from admission. On the third day of hospitalization, nasal oxygen inhalation with 1 L/min was needed due to a temporary decrease in SpO_2_, but quickly improved and could discontinue O_2_ inhalation on the same day. Despite a good general condition, his low-grade fever fluctuated around 37.5 °C and took 11 days to resolve. He was discharged on the 15th day from admission, 18 days after the onset of COVID-19 symptoms.

**Fig. 1:**
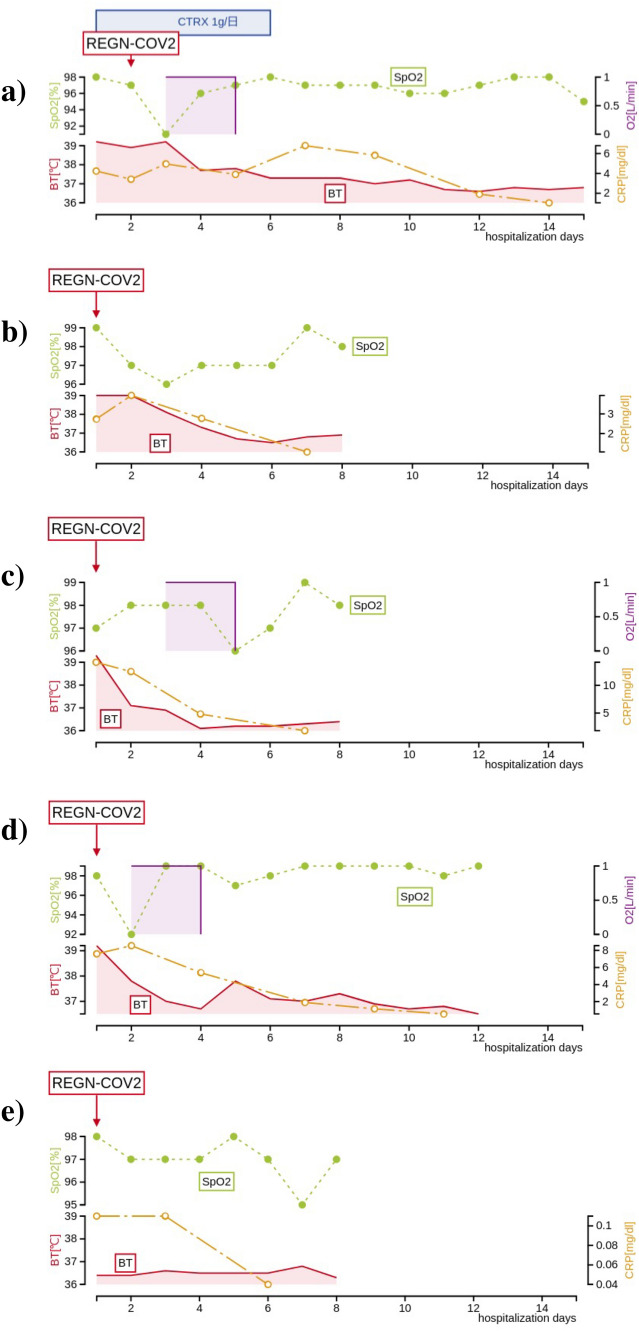
Clinical course of a patients treated with REGN-COV2 during hospitalization. The horizontal axis represents the time course of hospitalization (days). The solid line represents the patient's body temperature (°C), the dashed line represents the SpO_2_ (%), the closed circle represents the CRP value (mg/dl), and the shaded area indicates the oxygen inhalation period, respectively. **a** Case 1 (55-year-old man); CTRX 1 g/day was administered from the first day to the fifth day of admission, and REGN-COV2 was administered on the second day. The oxygen inhalation was required temporarily, but was terminated because of the quick improvement. His discharge date was 15 days after hospitalization. **b** Case 2. (50-year-old man); REGN-COV2 was administered on the day of admission, and his fever resolved on the 5th day after admission, with a concomitant decrease in CRP. He was discharged on the eighth day after admission. **c** Case 3 (78-year-old man); REGN-COV2 was administered on the day of admission, Oxygen was administered for about 24 h, and the fever improved with a decrease in CRP. He was discharged 8 days after admission. **d** Case 4 (71-year-old man); REGN-COV2 was administered on the day first of admission. The fever fluctuated until the 9th day after admission, the oxygen inhalation was required for 24 h. He was discharged on the 12th day after admission. **e** Case 5 (80-year-old man); REGN-COV2 was administered on the day of admission, and did not develop fever and oxygen inhalation, and was discharged on the 8th day. *CTRX* ceftriaxone sodium

**Fig. 2:**
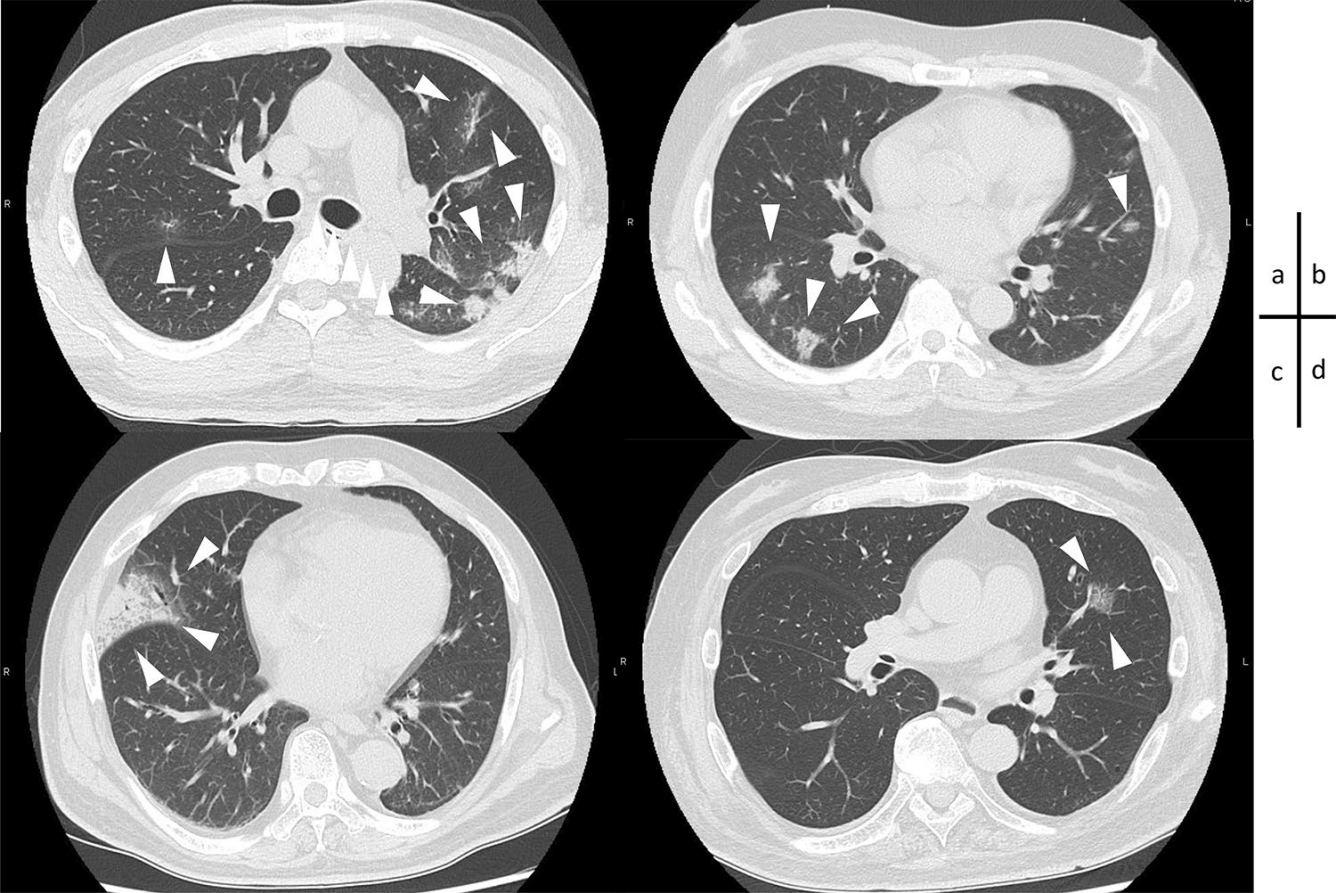
Chest HRCT images of patients treated with REGN-COV2 on admission (Case 1 to Case 4). Arrows indicate GGO considered to be caused by COVID-19. **a** Case 1; GGO bilaterally and infiltrative shadow with air bronchogram were shown in the left lower lung. **b** Case 2; multiple nodular to patchy opacities were revealed with peripheral predominance. **c** Case 3; GGO in the right middle lung. **d** Case 4; partial opacities located in the left lung. **d** Case 4; partial GGO in the left lung. *HRCT* high-resolution computed tomography, *GGO* ground

Case 2 (Fig. 1b) is a 50-year-old man, who was admitted to our hospital on persistent fever and cough. Nine days prior to admission, his daughter living with him, presented a high fever and diagnosed with COVID-19. He developed a fever of 37.6 °C and was also diagnosed with COVID-19 by PCR positive on the next day. He rested at home, but his fever worsened to 42 °C and was accompanied by nausea and vomiting. His hospital admission was on the seventh day after the initial symptoms. He had been on hemodialysis for approximately 8 years due to ESKD caused by DMN and had hypertension and glaucoma (blindness in the left eye). He was at risk for severe COVID-19 because of CKD grade 5, hypertension, and a history of smoking. He had received just one shot of vaccine 10 days before admission. On admission, his vital signs were followed: body temperature 39.0 °C, blood pressure 153/93 mmHg, heart rate 93 bpm, respiratory rate 22 /min, and SpO_2_ 99% without oxygen inhalation. On physical examination, there was no obvious abnormality in his breath sound, but CT scanning revealed multiple patchy opacities in bilateral peripheral lung (Fig. 2b). On the same day of hospitalization, he was administrated with REGN-COV2. He did not require oxygen inhalation throughout the hospitalization and had fever resolution on the fifth day after admission. He was discharged on the day eighth from the admission, 14 days after the onset of initial symptoms.

Case 3 (Fig. 1c) is a 78-year-old man, who was admitted to our hospital due to fever and cough. He met a close friend who had been previously diagnosed with COVID-19. Four days later, he developed a fever of 39.0 °C, cough, and sore throat, thus receiving PCR test and revealing positive. He had been recuperating at home for three days, but without improvement, he was admitted to our hospital. He had been on hemodialysis for 1 year and 5 months due to ESKD caused by DMN, and complicated hypertension, binocular glaucoma, and benign prostatic hyperplasia. He had a clinical history of angina pectoris and duodenal ulcer. His risks for severe COVID-19 were old age, CKD, type 2 DM, hypertension, and ex-smoker. He had already received the second shot of Corona-SARS-2 vaccine 56 days earlier. On admission, his body temperature was 39.3 °C, blood pressure 162/66 mmHg, heart rate 66/min, respiratory rate 23/min, and SpO_2_ 96% at room air. Physical examination revealed coarse crackles in the right lower chest, and CT scanning showed GGO in the right lung field (Fig. 2c). His CRP was markedly elevated at 17 mg/dl, but he did not require oxygen inhalation at the time of admission. REGN-COV2 was administered on the same day of admission. Although his fever resolved on the third day of hospitalization, he required a nasal oxygen due to mild hypoxia. O2 saturation was quickly improved on the next day, and was discharged on the eighth from the admission, 10 days after the first symptom.

Case 4 (Fig. 1d) is a 71-year-old man, who was admitted to our hospital because of fever, cough, and headache. The patient's son living with him, developed high fever, and diagnosed with COVID-19 by PCR test. He had also been aware of having a fever of 37.6 °C, and received the PCR test, and showed positive. He has been on hemodialysis for 11 years and 10 months due to ESKD caused by DMN, and has comorbidities such as hypertension, cataracts, and paroxysmal atrial fibrillation. He has a clinical history of cholecystectomy and appendicectomy. He was at high risk for severe COVID-19 because of his aging, CKD, type 2 DM and hypertension. On admission, his vital signs were normal except fever (39.2 °C) and high blood pressure (147/81 mmHg). His oxygen saturation was normal (SpO_2_ 98% at room air). Physical examination revealed no significant abnormality (Fig. 2d). CT scanning revealed a small consolidation in the left middle lung (Fig. 2d). REGN-COV2 was administered on the day of admission. On the second day of admission, he developed mild hypoxia and temporarily required oxygen with 2 L/min via nasal canula, but improved quickly on the same day and oxygen administration was discontinued. He also had an intermittent fever and became afebrile on the nineth day of hospitalization. He was discharged on the day 12th of hospitalization (two weeks after the initial symptoms).

Case 5 (Fig. 1e) is an 80-year-old man, who was admitted to our hospital due to nasal discharge and chills. His eldest son living with him, had been diagnosed with COVID-19 confirmed by PCR test, and the patient developed nasal discharge three days later. The next day, he underwent PCR test which revealed positive, and he was admitted to our hospital. He had been on hemodialysis for about a year due to ESKD with DMN. He had comorbidities such as hypertension, hyperuricemia, atrial fibrillation, and chronic heart failure. Risk factors for severe COVID-19 included high age, CKD, type 2 DM, hypertension, and ex-smoker. He completed the second vaccination 56 days earlier. On admission, his vital signs were normal, and he had no hypoxia. Physical examinations were normal, and there was no abnormality in Chest CT scan. Considering that the patient was at high risk of severe COVID-19, especially at an advanced age, REGN-COV2 was administered on the day of admission. After treatment, all symptoms disappeared on the second day of hospitalization, and he was afebrile and had never developed hypoxia throughout his hospitalization period. He was discharged home on the eighth hospitalization day (ten days after the onset of illness) (Table 1) .

**Table 1:** xxxx

Age, sex	Duration of HD treatment	Primary disease	Findings and results on admission					Tx	Admission day from onset (days)	Duration of hospitalization (days)	Maximum O_2_ inhalation during hospitalization	Outcome	Comorbidities	Smoking	Vaccination (times)
	(years, month)		Severity^a^	BT (℃)	SpO_2_ (%)	CRP (mg/dl)	HRCT								
55, M	8y, 10 m	DMN	Moderate I	39.2	98	4.24	GGO ( +)	REGN-COV2, CTRX	4	15	Nasal canula 1 L/min	IMP	DM, HT	( +)	(−)
50, M	7y, 11 m	DMN	Moderate I	39	99	2.74	GGO ( +)	REGN-COV2	7	8	(−)	IMP	DM, HT, glaucoma	( +)	× 1
78, M	1y, 5 m	DMN	Moderate I	39.3	96	14.17	GGO ( +)	REGN-COV2	3	8	Nasal canula 1 L/min	IMP	DM	ex	× 2
													HT, glaucoma,		
													BPH		
71, M	11y,10 m	DMN	Moderate I	39.2	98	7.6	GGO ( +)	REGN-COV2	3	12	Nasal canula 2 L/min	IMP	DM	(−)	× 2
													HT, glaucoma		
													,pAf		
80, M	1y, 2 m	DMN	Mild	36.4	98	0.11	(−)	REGN-COV2	3	8	(−)	IMP	DM, HT, pAf	ex	× 2
													Hyperuricemia, CHF		
38, F	2 m	ADPKD	Moderate II	38.3	100^b^	2.1	GGO ( +)	DEX	2	13	Nasal canula 1 L/min	IMP	HT	(−)	(−)
71, M	4y, 9 m	CGN	Moderate I	39.3	95	13.75	GGO ( +)	DEX	2	36	Mechanical ventilation (FiO_2_ 0.6)	IMP	pAf, HT	( +)	(−)
													OMI		
													CHF		
76, M	14y,0 m	DMN	Moderate I	37.5	98	21.6	GGO ( +)	VCM,CFPM	1	15	(−)	Transfer	DM, HT	ex	(−)
98, F	6y, 4 m	NSC	Moderate I	37.1	93	11.75	GGO ( +)	(−)	2	12	(−)	IMP	HT, OA	(−)	(−)
56, M	25y, 7 m	CGN	Moderate I	37	97	3.69	GGO ( +)	DEX	2	18	Nasal 1 L/min	IMP	HT, OMI	(−)	(−)
75, F	9y, 0 m	ADPKD	Moderate I	38.5	99	8.83	GGO ( +)	DEX	5	18	NHF (FiO_2_ 80% 40 L)	Dead	HT, DVT	(−)	(−)
86, F	1y, 2 m	DMN	Moderate I	37.5	95	16.42	GGO ( +)	SBT/ABPC	4	19	(−)	Transfer	DM, HT, RA,	(−)	(−)
													ASO, dementia		
82, F	8 m	AAV	Moderate I	36.6	96	2.91	GGO ( +)	(−)	7	26	Nasal 1 L/min	IMP	AAV, DM	(−)	(−)
													pAf, BA		
78, M	6y, 1 m	NSC	Moderate I	37.5	96	4.07	GGO ( +)	DEX	7	21	Nasal 3 L/min	IMP	HT	ex	(−)
													Nephrectomy		
45, M	6y, 9 m	DMN	Moderate I	37.7	97	3.83	GGO ( +)	DEX	9	22	Nasal 1 L/min	IMP	DM, HL, HT	ex	(−)
54, M	7y, 4 m	DMN	Moderate I	37.7	95	10.84	GGO ( +)	DEX	6	14	Mask 4 L/min	IMP	DM, HT,	(−)	(−)
74, F	8y, 6 m	DMN	Mild	36.8	98	0.09	(−)	(−)	9	9	Nasal 1 L/min	IMP	DM, HT, HL	(−)	(−)
63, F	2y, 0 m	DMN	Mild	36.4	98	0.15	(−)	(−)	1	11	(−)	IMP	DM, HT	ex	(−)
71, M	5y, 4 m	IgAN	Moderate II	39.4	96 ^b^	16.24	GGO ( +)	DEX,CTRX	4	13	Nasal 3 L/min	IMP	HT, HL, hyperuricemia	(−)	× 2
90, F	7 m, 0 m	DMN	Moderate I	37.8	97	2.24	GGO ( +)	DEX,CTRX	5	23	Nasal 1 L/min	Transfer	DM, OMI, HT, Af	(−)	× 2
57, M	0 m	ADPKD	Moderate I	37.9	96	0.62	GGO ( +)	DEX, TOC	1	30	NHF(FiO_2_ 75% 40 L)	Transfer	HT	(−)	× 1
64, M	8y, 3 m	TIN	Moderate I	38.2	100	8.53	GGO ( +)	DEX, CTRX, MEPM, CAZ	5	76	Intubation(FiO_2_ 60)	Dead	HT, BPH	(−)	(−)

*DMN* diabetic nephropathy; *ADPKD* autosomal dominant polycystic kidney disease; *CGN* chronic glomerulonephritis; *NSC* nephrosclerosis; *AAV* ANCA-associated vasculitis; *IgAN* IgA nephritis; *HRCT* high-resolution computed tomography; *TX* treatment; *CTRX* ceftriaxone sodium; *DEX* dexamethasone; *VCM* vancomycin; *CFPM* cefepime; *SBT/ABPC* sulbactam/ampicillin; *TOC* tocilizumab; *MEPM* meropenem; *CAZ* ceftazidime; *DM* diabetes mellitus; *HT* hypertension; *BPH* benign prostatic hypertrophy; *pAf* periodic atrial fibrillation; *CHF* chronic heart failure; *OMI* old myocardial infarction; *OA* osteoarthritis; *DVT* deep vein thrombosis; *RA* rheumatoid arthritis; *ASO* atherosclerosis obliterans; *AAV* ANCA-associated angiitis; *OMI* old myocardial infarction; *BA* bronchial asthma; *HL* hyperlipemia

^a^The notation of disease severity followed the Japanese diagnostic guide[8]; “mild disease” refers to cases without complications of pneumonia, “Moderate I” refers to cases with pneumonia but no need for oxygen inhalation, “Moderate II” refers to cases with pneumonia and need for oxygen inhalation, “Severe” refers to cases that require a ventilator or nasal high flow (NHF)

^b^This patient was receiving nasal oxygen inhalation at 1 L/min

### Comparison between REGN-COV2 treated and non-treated patients

Table 2 summarized the cases of maintenance hemodialysis patients admitted to our hospital for COVID-19 since March 2020. The total number of patients was 22, including three mild cases without pneumonia and decreased oxygenation, 17 cases with pneumonia but not requiring oxygenation at the time of admission (Severity class: moderate I), and two cases with pneumonia and decreased SpO_2_ (Severity class: moderate II). All but four patients had fever above 37 °C. The duration of hospitalization was 19.41 ± 15.28 days (mean ± SD), median duration of hospitalization was 15 days (minimum 8 days to maximum 76 days) (*P* = 0.0553), most patients were discharged after recover or better illness, and there were two deaths. Since the indication for administration of REGN-COV2 is defined in Japan as cases within 7 days of onset and up to severity class of moderate I, 17 cases who were hospitalized before the approval of this medication did receive REGN-COV2 under the same conditions (20 cases) were used as the control group “REGN-COV2(−)” and compared with five patients who received REGN “REGN-COV2( +)” (Table 2). In this setting, the average CRP on admission for cases in the REGN-COV2(−) group was 7.28 ± 6.51 mg/dl (mean ± SD) and the duration from onset to admission was 4.4 ± 2.82 days. In contrast, five “REGN-COV2( +)” patients showed an average CRP of 5.77 ± 5.41 mg/dl on admission and a duration from onset to their admission of 4.0 ± 1.73 days. There was no statistically significant difference between these two groups. Post-hospitalization course and outcomes in the REGN-COV2(−) and REGN-COV2( +) groups were as follows, the hospitalization period was 23.33 ± 16.28 days vs. 10.2 ± 3.19 (*P* = 0.047), oxygen inhalation during hospitalization was 11 out of 15 (73.3%) vs. 3 out of 5 (60%) and 2 died (13.3%) vs. none, respectively. The maximum hospitalization period for the REGN-COV2( +) was 15 days, which was much shorter than the average of the non-treated group. The mean duration of fever in the non-treated patients was 160.86 ± 128.72 h, compared to 110.0 ± 84.2 h in the REGN-COV2 treated patients (*P* = 0.443). As for the administration procedure, all patients came to the hospital unrelated to dialysis and were administered REGN-COV2 if patients fulfilled the criteria for its administration indication. Although the antibody cocktail is a polymeric formulation, no volume over-loading was observed. Thus, it was not necessarily to consider the timing of dialysis and its administration. None of the patients who were treated with REGN-COV2 received dexamethasone during their hospitalization, despite nine out of 15 patients in the non-administration group were received dexamethasone because of severity of COVID-19.

**Table 2:** Patient who received REGN-COV2 vs patients who did NOT receive REGN-COV2

	REGN-COV2 ( +)	REGN-COV2 (−)
Number of patients	5	15
Age	66.8 ± 12.15	71.26 ± 14.21
HD duration	6.23 ± 4.23	7.64 ± 5.83
Average CRP value on admission (mg/dl)	5.77 ± 4.85	7.51 ± 6.42
Hospitalization periods (days)	10.2 ± 2.86*	22.12 ± 15.15
Oxygen inhalation (case)	3 out of 5 (60%)	13 out of 15 (86.6%)
Duration of fever (h)	110.0 ± 84.2	160.86 ± 128.72
Dexamethasone use during hospitalization	None	9 out of 15 (60.0%)
Dead (case)	None	2 (13.3%)

A comparison was made between patients with mild to moderate disease who are eligible for REGN-COV2. Each values are represented as mean ± SD

^*^P < 0.05 by unpaired study *t* test

## Discussion

We treated five hemodialysis patients affected by COVID-19 with REGN-COV2. In all cases, early improvement was observed, and the hospitalization periods could be also shortened.

The first case of COVID-19 in a hemodialysis patient in Japan was reported on March 1st, 2020. Since then, the number of hemodialysis patients infected with SARS-CoV-2 has increased in accordance with the rapid spread of COVID-19 in the general population [6]. ESKD patients with hemodialysis has been considered to be at high risk for severe COVID-19 because of their impaired immunity and many comorbidities [7]. In Japan, remdesivir has been proposed for the treatment of symptomatic patients that do not require oxygen inhalation in non-dialysis patients, and glucocorticoids and baricitinib for patients with moderate-severe, pneumonia who require oxygen inhalation. It has been suggested that remdesivir can increase the recovery rate, decrease mortality, and shorten the disease period of in adults with severe COVID-19 [8, 9]. However, the use of remdesivir and baricitinib to the patients with ESKD is not recommended in Japan, but rather contraindicated, because of the rapid increase in its serum concentration. For this reason, only glucocorticoid therapy is available for the ESKD patients with hemodialysis. Therefore, the high incidence of severe disease, and the lower survival rate may have been shown in these patients [10], and early administration of medication that can be expected to prevent severe disease is desirable.

On July 19, 2021, REGN-COV2, which is a combined neutralizing-monoclonal antibodies targeting the receptor-binding domain of the spike protein of SARS-CoV-2, was granted special approval in Japan. In U.S. Phase III clinical trials, a single dose of REGN-COV2 was shown to reduce the severity of the disease and the overall duration of illness, and the drug is expected to be effective in Japan [11]. It has also been reported to be effective against virus strains with mutations in the spike protein [12]. At present (August 27, 2021) in Japan, patients who are age 65 years or older, have malignant tumors, chronic obstructive pulmonary disease, CKD, hypertension, dyslipidemia, obesity with a BMI of 30 or higher, smoker and ex-smoker, immunodeficiency after solid organ transplantation, or late pregnancy are defined to be at risk for severe disease. Patients who meet one or more of these conditions and are judged to have mild to moderate disease that does not require oxygen inhalation are considered to be eligible for REGN-COV2. According to the indication criteria, all hemodialysis patients are considered to be at risk for severe disease.

Our clinical experience suggested that treatment with REGN-COV2 can shorten the hospitalization days by about half compared to patients with comparable disease. It also prevented severe disease during the hospitalization period, and none of the cases resulted in death. This strongly suggested that the drug may be an effective treatment to prevent serious illness in dialysis patients. Three of the cases deteriorated to the point where oxygen was required despite the administration of REGN-COV2, but none of them became more severe and could be discharged without requiring mechanical ventilation. Case 1 had the longest hospitalization period, 15 days. He had a complication of bacterial infection and had never been vaccinated, suggesting he did not have antibodies against SARS-CoV-2. Case 3 was the oldest patient and had a high CRP level of 14.17 mg/dl, but his fever resolved 2 days after the treatment, and SpO2 also improved on the next day. The severity of pneumonia in this case was less extensive compared to the other two cases, and the CRP in dialysis patients with this disease may have indicated that their immune status may be relatively well maintained. Case 4 had been vaccinated and REGN-COV2 had been administered earlier from the onset, but his hospitalization period was prolonged to 12 days. He had been on dialysis for the longest time (almost 12 years) among them, thus the duration of dialysis therapy could be a risk factor for severe disease among hemodialysis patients. Case 5, who was mildly ill at the time of admission, was discharged without worsening of symptoms and without the need for oxygen administration. He was the oldest of these cases, and it is likely REGN-COV2 was a safe and effective treatment for the elderly ESKD with hemodialysis.

There are several reasons why this cocktail therapy is not only feasible but also of great benefit in maintenance hemodialysis patients. First, maintenance hemodialysis patients are seen three times a week for hemodialysis visits, which makes it easier to detect the onset of symptoms such as fever and cough, and to diagnose COVID-19 promptly after the onset of symptoms because they have good access to antigen and PCR tests. In other words, it is easy to find early-onset cases for which REGN-COV2 is indicated. Secondly, because of the risk of complications such as anaphylaxis with this drug, hospitalization or several hours observation is necessary, physical observation can be performed safely and easily since hemodialysis treatment is generally lasted for more than 4 h in the clinic. Third, infectious disease is among the leading causes of morbidity and mortality in patients with underlying ESKD [13, 14]. Besides the vaccination is also reported less effective in dialysis patients [15, 16]. Neutralizing antibody supplementation can complement the patient's own antibody titer against SARS-CoV-2. Dialysis facilities are likely to lead to the spread of COVID-19 because patients are treated in open areas for long periods of time, rather early intervention of REGN-COV2 is necessary.

## Conclusion

We treated five hemodialysis patients affected by COVID-19 with REGN-COV2. In all cases, early improvement was observed, and the hospitalization periods could be also shortened. It was suggested that REGN-COV2 could be a key drug to prevent severe disease in hemodialysis patients, who had limited treatment options. In addition, preventing severe disease with the use of this medication would be beneficial in terms of limited medical resources and hospital bed management among ESKD patients with hemodialysis.

### Limitations

The location and population characteristics of those treated at Kyorin University Hospital may not allow these results to be generalizable across all patient populations in ESKD patients treated with hemodialysis.

## Data Availability

All data generated or analyzed data were obtained from Kyorin University Hospital and are included in this published article.

## Abbreviations

SARS-CoV2: Severe acute respiratory syndrome coronavirus 2
ESKD: End-stage kidney disease
DM: Diabetes mellitus
DMN: Diabetic nephropathy
GGO: Ground glass opacities
